# Omega-3 Polyunsaturated Fatty Acids Time-Dependently Reduce Cell Viability and Oncogenic MicroRNA-21 Expression in Estrogen Receptor-Positive Breast Cancer Cells (MCF-7)

**DOI:** 10.3390/ijms19010244

**Published:** 2018-01-14

**Authors:** Lauren LeMay-Nedjelski, Julie K. Mason-Ennis, Amel Taibi, Elena M. Comelli, Lilian U. Thompson

**Affiliations:** 1 Department of Nutritional Sciences, Faculty of Medicine, University of Toronto, Toronto, ON M5S 1A8, Canada; l.lemay@mail.utoronto.ca (L.L.-N.); julie.mason@mail.utoronto.ca (J.K.M.-E.); amel.taibi@utoronto.ca (A.T.); elena.comelli@utoronto.ca (E.M.C.); 2 Joannah and Brian Lawson Centre for Child Nutrition, Faculty of Medicine, University of Toronto, Toronto, ON M5S 1A8, Canada

**Keywords:** breast cancer, n-3 PUFAs, α-linolenic acid, eicosapentaenoic acid, docosahexaenoic acid, microRNA-21

## Abstract

The omega-3 polyunsaturated fatty acid (n-3 PUFA), α-linolenic acid (ALA), and its metabolites, eicosapentaenoic acid (EPA) and docosahexaenoic acid (DHA), independently reduce the growth of breast cancer cells in vitro, but the mechanisms, which may involve microRNA (miRNA), are still unclear. The expression of the oncomiR, miR-21, is reduced by DHA treatment, but the effects of ALA on miR-21, alone or combined with EPA and DHA under physiologically relevant concentrations, have not been investigated. The effects of ALA alone and +/−EPA and DHA at the blood molar ratios seen in either humans (1.0:1.0:2.5, ALA:EPA:DHA) or mice (1.0:0.4:3.1, ALA:EPA:DHA) post flaxseed oil consumption (containing ALA) were assessed in vitro in MCF-7 breast cancer cells. Cell viability and the expression of miR-21 and its molecular target, phosphatase and tension homolog (PTEN, gene and protein), at different time points, were examined. At 1, 3, 48 and 96 h ALA alone and 24 h animal ratio treatments significantly reduced MCF-7 cell viability, while 1 and 3 h ALA alone and human and animal ratio treatments all significantly reduced miR-21 expression, and 24 h animal ratio treatment reduced miR-21 expression; these effects were not associated with changes in PTEN gene or protein expressions. We showed for the first time that ALA alone or combined with EPA and DHA at levels seen in human and animal blood post-ALA consumption can significantly reduce cell viability and modulate miR-21 expression in a time- and concentration-dependent manner, with the animal ratio containing higher DHA having a greater effect. The time dependency of miR-21 effects suggests the significance of considering time as a variable in miRNA studies, particularly of miR-21.

## 1. Introduction

Breast cancer is the most common form of cancer in women worldwide with an estimated 1.8 million new cases and approximately 471,000 deaths in 2013 [1,2]. Targeted breast cancer treatment protocols based on molecular subtypes are available, which have greatly improved outcomes; however, treatment side effects and resistance remain important clinical issues. Many patients and clinicians are turning towards complementary medicine, including functional foods, to improve the effectiveness and tolerability of conventional treatments [3]. These include flaxseed (FS; *Linum usitatissimum*) and fish oil (FO), two sources of omega-3 polyunsaturated fatty acids (n-3 PUFA) [4].

Flaxseed contains oil (FSO) is rich in the n-3 PUFA, α-linolenic acid (ALA; 18:3n-3), while FO is rich in the n-3 PUFAs eicosapentaenoic acid (EPA; 20:5n-3) and docosahexaenoic acid (DHA; 22:6n-3) [5,6]. ALA is the metabolic precursor of EPA and DHA, and consumption of ALA or ALA-rich foods by healthy humans or animals results in elevations of blood concentrations of ALA, EPA and to some extent, DHA [5,7]. In humans, a blood molar ratio of 1.0:1.0:2.5, ALA:EPA:DHA has been recorded following consumption of 2 g of FSO in capsule form; similarly, in mice, a molar blood ratio of 1.0:0.4:3.1, ALA:EPA:DHA has been observed following a 40 g/kg FSO supplemented basal diet [5,7]. Therefore, when ALA is consumed, it is not clear whether the observed effects are due to ALA alone, its EPA and DHA metabolites, or their combination. In vitro studies are useful in this context since mammalian breast cancer cells lack the necessary desaturase enzyme to convert ALA to EPA and DHA [8]. In vitro, ALA, EPA and DHA have all been shown to independently reduce the growth of breast cancer cells and induce apoptosis [5,9,10]. However, the mechanisms underlying n-3 PUFA specific effects remain unclear, particularly at the post-transcriptional level via microRNA (miRNA). To date, no in vitro studies have looked at the miRNA-mediated response of breast cancer cells to ALA alone or ALA:EPA:DHA combination at molar blood ratios observed previously in vivo post-ALA consumption [5].

MiRNA-21 (miR-21) is an oncomiR found to be overexpressed in the serum and breast tissue of breast cancer patients compared to healthy controls [11,12,13,14,15]. MiR-21 has many validated molecular targets, however, the work of Mandal and colleagues [16] focusing on DHA, miR-21 and PTEN has prompted our group to take a closer look at n-3 PUFAs, miR-21 and one of its targets, phosphatase and tension homolog (PTEN) [16]. MiR-21 promotes the growth and proliferation of breast cancer cells in vitro, and tumor growth in nude mice in vivo via its association with its target, PTEN [16,17]. DHA reduces miR-21 and increases PTEN expression, but the effect of ALA alone or in combination with its metabolites on miR-21 remains unknown [16,17]. MiRNAs are dynamic molecules, as their expression, as well as their gene and protein targets, change rapidly and repeatedly over time [18]. Thus, temporal effects should be considered as a variable in miRNA studies.

This study aimed to determine the effect of ALA alone and combined with EPA and DHA at the blood molar ratios seen in either humans (1.0:1.0:2.5, ALA:EPA:DHA) or animals (mice) (1.0:0.4:3.1, ALA:EPA:DHA) post FSO consumption (containing ALA) on MCF-7 cell viability, miR-21 and one of its targets, PTEN expression.

## 2. Results

### 2.1. Effect of ALA Alone or Combined with EPA and DHA on Cell Viability after 1, 3, 24, 48 and 96 h Treatment

**Figure 1:**
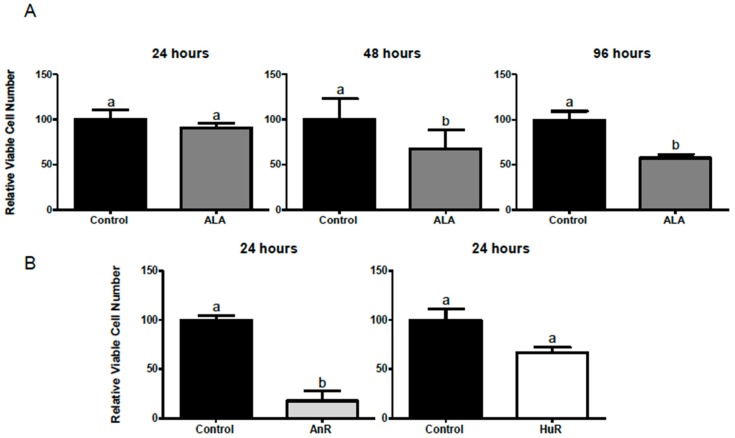
(**A**) Effect of 100 μM ALA on cell viability after 24, 48 and 96 h treatment. No difference was seen after 24 h of treatment, but significantly fewer total live cells were found after 48 (*p* < 0.005) and 96 h (<0.05); (**B**) Effect of ALA combined with EPA and DHA on cell viability after 24 h treatment. The AnR (25 μM ALA:9 μM EPA:78 μM DHA) treatment resulted in significantly fewer live cells compared to control (82.2% reduction; *p* < 0.01), while the HuR (25 μM ALA:25 μM EPA:62 μM DHA) treatment had no significant effect compared to the control *. * Cell viability is expressed as a % of the control viable cell number. Bars with different letters (a,b) are significantly different from one another (*p* < 0.05). AnR = animal fatty acid ratio; HuR = human fatty acid ratio; ALA = α-linolenic acid.

ALA (100 μM) alone significantly reduced the viability of MCF-7 cells after 48 or 96 h treatment, but not 24 h (Figure 1). MCF-7 cells were then treated with ALA, combined with EPA and DHA, at ratios observed post-FSO consumption. The animal ratio treatment (animal fatty acid ratio (AnR); 25 μM ALA:9 μM EPA:78 μM DHA) significantly reduced cell viability (82.2% ± 10.3 reduction; *p* ≤ 0.01), while the human ratio treatment (human fatty acid ratio (HuR); 25 μM ALA:25 μM EPA:62 μM DHA) non-significantly reduced cell viability (Figure 1). Insufficient cells remained with AnR treatment beyond 24 h and HuR at 48 h to permit further measurement of cell viability.

### 2.2. Effect of ALA Alone or Combined with EPA and DHA on miR-21 Expression at Different Time Points 

**Figure 2:**
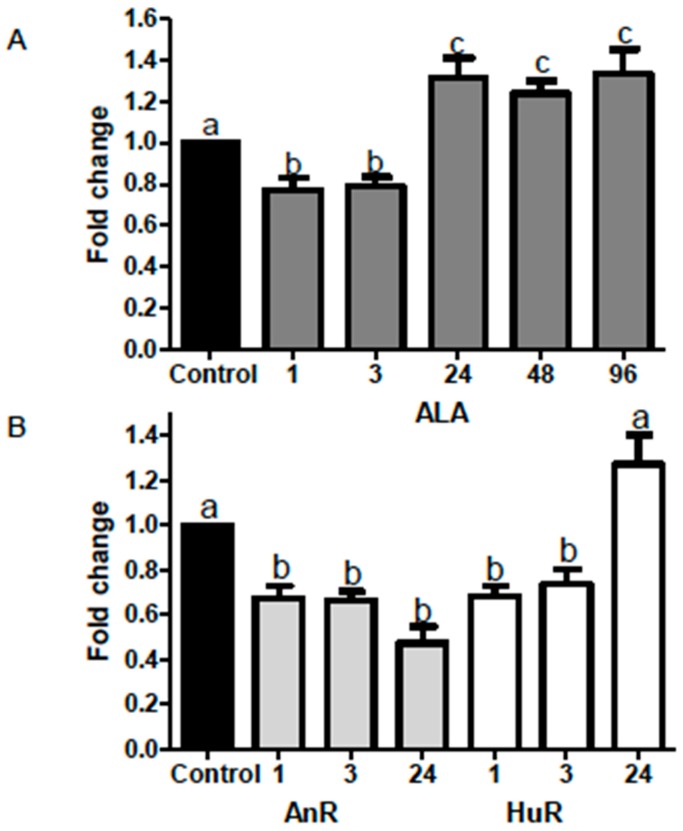
(**A**) Effect of ALA on miR-21 expression after 1, 3, 24, 48 and 96 h treatment (dark grey). ALA alone significantly reduced miR-21 expression following both 1 and 3 h treatment (fold changes of 0.77 ± 0.05 and 0.79 ± 0.45 respectively, *p* < 0.05). MiR-21 was then significantly increased following the same treatment for 24 (30%; fold change = 1.3 ± 0.09; *p* < 0.05), 48 (20%; fold change = 1.2 ± 0.06); *p* < 0.01) and 96 h (30%; fold change = 1.3 ± 0.12; *p* < 0.05) compared to controls *; (**B**) Effect of ALA combined with EPA and DHA on miR-21 expression after 1, 3 and 24 h treatment. The AnR (25 μM ALA:9 μM EPA:78 μM DHA) (light grey bars) and HuR (25 μM ALA:25 μM EPA:62 μM DHA) (white bars) treatments both significantly reduced miR-21 expression after 1 and 3 h (fold changes AnR = 0.67 ± 0.05–0.66 ± 0.04; HuR = 0.68 ± 0.04–0.74 ± 0.07; *p* < 0.001). At 24 h, AnR also significantly reduced miR-21 (fold-change of 0.5 ± 0.007; *p* < 0.001), but HuR had no effect when compared to control *. * Data were normalized to the endogenous control U6 and fold change was calculated with the 2^−ΔΔ*C*t^ method [19]. Bars with different letters (a,b,c) are significantly different from one another (*p* < 0.05). AnR = animal fatty acids ratio; HuR = human fatty acids ratio.

ALA (112 μM) (see * Note in Materials and Methods) alone significantly reduced miR-21 expression after both 1 and 3 h treatment (fold changes ALA = 0.77 ± 0.05 and 0.79 ± 0.45 respectively, *p* < 0.05). Conversely, miR-21 expression was significantly increased following 24, 48 and 96 h treatment (30%; fold change = 1.3 ± 0.09; *p* < 0.05; 20%; fold change = 1.2 ± 0.06); *p* < 0.01; 30%; fold change = 1.3 ± 0.12; *p* < 0.05) (Figure 2).

ALA combined with EPA and DHA in AnR and HuR significantly reduced miR-21 expression after both 1 and 3 h treatment (fold changes AnR = 0.67 ± 0.05–0.66 ± 0.04; HuR = 0.68 ± 0.04–0.74 ± 0.07; *p* < 0.001). miR-21 expression was also significantly downregulated following 24 h treatment with the AnR (fold-change = 0.5 ± 0.007; *p* < 0.001) when compared to the control. No significant changes in miR-21 expression were observed after 24 h of treatment with the HuR (Figure 2).

### 2.3. Effect of ALA Alone or Combined with EPA and DHA on PTEN Gene and Protein Expression after 12 and 24 h Treatment 

**Figure 3:**
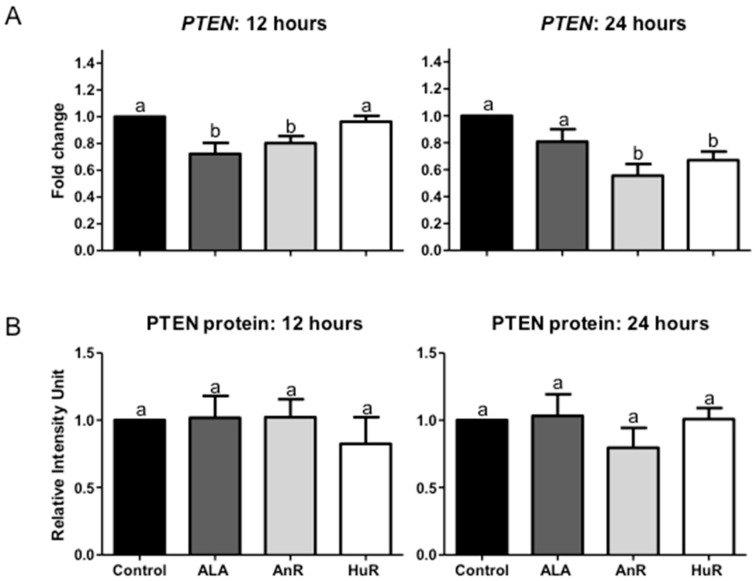
(**A**) Effect of ALA alone and combined with EPA and DHA on *PTEN* gene expression. 112 μM ALA and AnR significantly reduced *PTEN* gene expression following 12 h treatment (ALA fold change = 0.72 ± 0.09, AnR fold change = 0.80 ± 0.1; *p* < 0.01); no change was seen with HuR treatment. Following 24 h treatment, AnR and HuR significantly reduced *PTEN* gene expression (AnR fold change = 0.55 ± 0.08; HuR fold change = 0.67 ± 0.06; *p* < 0.001); no change was observed with ALA treatment. Bars with different letters are significantly different (*p* < 0.05). Data were normalized to the endogenous control beta-2 microglobulin (B2M) and fold change was calculated with the 2^−ΔΔ*C*t^ method [19]. AnR = animal fatty acids ratio; HuR = human fatty acids ratio; (**B**) Effect of ALA alone and combined with EPA and DHA on PTEN protein expression. No change in PTEN protein expression was observed following both 12 and 24 h treatment of 100 μM ALA, AnR and HuR. GAPDH was used as the loading control. Bars with different letters (a,b) are significantly different from one another (*p* < 0.05). AnR = animal fatty acids ratio; HuR = human fatty acids ratio.

ALA (112 μM) alone and AnR significantly reduced *PTEN* gene expression following 12 h treatment (ALA fold change = 0.72 ± 0.09, AnR fold change = 0.80 ± 0.1; *p* < 0.01); no change was seen with HuR treatment (Figure 3). Following 24 h treatment, AnR and HuR significantly reduced *PTEN* gene expression (AnR fold change = 0.55 ± 0.08; HuR fold change = 0.67 ± 0.06; *p* < 0.001); no change was observed with ALA treatment (Figure 3).

No difference in PTEN protein expression was observed following both 12 and 24 h treatment among the treatment groups and the control (Figure 3).

## 3. Discussion

This study is the first to address the time- and n-3 PUFA concentration-dependent nature of cell viability and miR-21 expression in human breast cancer cells. We showed that miR-21 expression is significantly decreased following shorter treatment periods (1 and 3 h), regardless of n-3 PUFA treatment (ALA +/− EPA + DHA at AnR or HuR) but this was not associated with changes in its validated molecular target, PTEN, at either the gene or protein level.

In the current study, treatment concentrations were modeled based on the relative blood levels of ALA, EPA and DHA observed after FSO interventions in mice and humans, where mice have been shown to have relatively more DHA and less EPA (ALA:EPA:DHA = 1.0:1.0:2.5 in human, versus 1.0:0.4:3.1 in mice) [5,7]. Kang et al., [20] showed that in MCF-7 cells, the IC_50_ values for DHA and EPA were 20.2 and 57.4 μM, respectively. Additional work with n-3 PUFAs has also shown that the IC_50_ values for the fatty acids are as follows: ALA > EPA > DHA, demonstrating that DHA possesses the greatest bioactive capacity, which enables DHA to most significantly impact cell death [21]; in line with this, the greatest effect in terms of cell viability reduction in our study was in the AnR treatment, which had the highest DHA concentration. The greater effect of DHA may be due to its ability to trigger an increased production of reactive oxygen species in breast cancer cells through its additional double bonds and larger size, which may subsequently lead to cell death via apoptosis [20].

Previous work conducted in our lab has shown that ALA is significantly incorporated into cellular phospholipids following in vitro ALA treatment, but formation of ALA metabolites, including EPA and DHA was not observed [8,9,22]. Accordingly, EPA and especially DHA have been found to be reduced in ALA-treated cells, as well as total omega-6 (n-6) PUFA and monounsaturated fatty acids being significantly lower [8,9,22]. Additionally, it has been shown that treatment with DHA increases phospholipid DHA in breast cancer cells [23,24,25]. The combination of the three n-3 PUFAs in the treatment medium would likely result in the fatty acids competing for incorporation into the cellular phospholipids and, thus each treatment ratio would result in a different phospholipid fatty acid profile. Incorporation of n-3 PUFAS into the cellular phospholipids would then alter membrane fluidity and permeability, as well as disrupt cholesterol-rich regions in the cell membrane known as lipid rafts [26,27]. Proteins involved in cell signal transduction such as receptor tyrosine kinases (RTKs), G-protein coupled receptors (GPCRs), G-proteins, kinases and phosphatases are found concentrated within these cholesterol and glycosphingolipid-rich lipid rafts [28,29,30]. Disruption of raft proteins, such as lateral movement of proteins from raft to non-raft domains, or changes in the fatty acid composition of rafts by n-3 PUFAs, can result in altered oncogenic signaling in breast cancer cells [28]. Two key membrane phospholipids that may be impacted by n-3 PUFA incorporation are phosphatidylinositol (4,5)-bisphosphate (PIP2) and phosphatidylinositol (3,4,5)-trisphosphate (PIP3) [31]. These are involved in activation of the PI3K/Akt pathway, which includes PTEN and is involved in cell growth [31]. This may be one explanation as to why ALA levels alone in this study cannot predict cell viability completely, as each ratio versus ALA alone brings about a new and competing combination of n-3 PUFAs to incorporate into the cellular phospholipids. Although the phospholipid fatty acid profile was not examined in this study, based on our previous work and the literature to date, it can be assumed that the n-3 PUFAs in the treatment medium did incorporate into the cellular phospholipids and are at least partially responsible for the observed reduction in cell viability.

The mechanism whereby n-3 PUFAs trigger a reduction in cell viability remains unknown; however, previous work with n-3 PUFAs and cancer cells have shown n-3 PUFAs not only to reduce cell viability, as was seen in this study, but also induce apoptosis selectively in cancer cells, while leaving normal cells unharmed [9,22,32]. ALA has been found to inhibit the growth of MCF-7 cells in vitro without cytotoxicity [33]. The doses used in this study are very moderate as in humans fed 2 g FSO or 2 g FO, plasma fatty acid profiles were seen to be 90 μM ALA, 84 μM EPA, 230 μM DHA, 484 μM total for the FSO group and, 73 μM ALA, 115 μM EPA, 290 μM DHA, 478 μM total for FO [7]. Similarly, in mice fed 40 g/kg/day pure FSO, the total amount was found to be 61 μM ALA + 22 μM EPA + 193 μM DHA = 276 μM [5]. Therefore, the total dose used in our study is not huge and well within physiological range in vivo. Doses as low as 50 μM ALA have been shown to significantly reduce cell proliferation, measured using a BrdU assay kit, when compared to untreated cells [5]. Additional work by our group on the effect of ALA on apoptosis induction measured using annexin V staining in MCF-7 cells showed no significant effect [8,9]. Due to these findings, it was hypothesized in [22] that for ALA the growth retardation is likely due to inhibition of proliferation and not induction of apoptosis. In contrast, n-3 PUFAs EPA and DHA have been shown to induce apoptosis via alteration of B-cell lymphoma 2 (Bcl-2) and procaspase-8 and reduce cell growth in both estrogen receptor-negative and positive breast cancer cells in vitro [22]. The doses of n-3 PUFAs used in this current study were not cytotoxic and a reduction in cell viability was observed.

The mechanisms of the n-3 PUFA effect on miRNA remain unknown. Mandal and colleagues previously showed that a very low dose of DHA alone was able to reduce miR-21 expression after 24 h via a mechanism involving nuclear factor kappa-B (NF-κB) [16]. The promoter of miR-21 contains an NF-κB-binding element and previous work has found miR-21 to be regulated by NF-κB [34]. Transfection experiments carried out by Mandal et al. [16] demonstrated that miR-21 transcription is at least partially mediated by NF-κB. The differential expression of miR-21 across varying time points and treatments may also be due to miRNAs other than miR-21, which may have either inhibited or augmented the n-3 PUFA effect on miR-21 [35,36,37,38]. ALA may be altering additional miRNAs other than miR-21 at later time points, which would interfere with or inhibit any effect it may have on its targets [35,36,37,38]. A recent in vivo study showed that mice injected with human colon cancer cells and fed walnuts, a rich source of ALA, displayed an altered miRNA expression profile (reduced miR-467c, −1903, −3068) paired with a reduction in tumor growth, supporting the hypothesis that ALA can modulate the expression of several miRNA other than just miR-21 [39]. This may further explain the lack of correlation observed between miR-21 and PTEN expression at various time points. If additional miRNA were interfering in the signaling or mechanism at play, this may disrupt downstream targets, such as PTEN, resulting in unexpected levels based on the miR-21 expression observed. Lastly, it cannot be excluded that ALA may be working through non-miRNA mechanisms in reducing cell viability, such as by inducing cell death, either by apoptosis or necrosis, or by interfering with cell cycle progression [38,40].

PTEN is a negative regulator of PI3K/Akt signaling and a prominent validated target of miR-21 in several cancers [16,41]. Perturbations in PTEN functionality have been repeatedly implicated in breast cancer pathogenesis and PTEN has previously been shown to be impacted by the n-3 PUFAs studied here, although the mechanism remains unknown [4,42,43,44]. Mandal et al. [16] also showed that *PTEN*, a validated target of miR-21, was upregulated in response to reduced miR-21 via DHA treatment. In our study, *PTEN* gene expression was reduced following both 12 and 24 h treatment with AnR, which juxtaposes the reduction in miR-21 seen at 1, 3 and 24 h after AnR. This may be due to competing fatty acid incorporation into the cellular phospholipids, paired with miRNAs other than just miR-21 also targeting or competing for PTEN or related proteins within the PI3K/Akt pathway. These findings also likely highlight a lag time that exists before the altered miR-21 expression has an effect on its PTEN gene and/or protein target; this has been observed for other miRNA and we may not have examined sufficient time points to pick on this change [41]. Finally, miR-21 may need to reach a sustained threshold magnitude fold-change reduction before it can alter gene expression of its target, which again, highlights the need to investigate miR-21 and PTEN at additional time points. Due to the discrepancy, these findings indicate that the overall mechanism of cell viability reduction likely does not occur via miR-21 modulation of PTEN and there are additional mechanisms of action at play.

The strength of this study lies in the novel n-3 PUFA treatment ratios and the multiple time points examined in terms of cell viability, miR-21 expression, and gene and protein biomarker expression. MiR-21 has been identified as a potential prognostic and diagnostic biomarker for breast cancer; thus, the time-dependent effects of a complementary treatment, such as n-3 PUFAs, may have important clinical implications [11,12,13,14,15]. Future time-course, transfection studies are needed to investigate the mechanisms involved, including the miR-21 targets, as well as in vivo investigations of the miR-21 time course. DHA appears to be a prominent bioactive n-3 PUFA in vivo, however, the ALA effect remains strong in vitro where no conversion occurs. Further research is required to elucidate an ALA-specific mechanism of action in breast cancer cells.

## 4. Materials and Methods

### 4.1. Cell Line, Cell Culture and Treatment Medium

The ER+, PR+ breast cancer cell line MCF-7, was purchased from American Type Culture Collection (ATCC, Manassas, VA, USA) and cultured in DMEM medium (Gibco, Carlsbad, CA, USA) supplemented with 10% fetal bovine serum (FBS; Sigma-Aldrich, St. Louis, MO, USA) and 1% antibiotic-antimycotic solution containing penicillin, streptomycin and amphotericin B (Gibco). MCF-7 cells were maintained in a humidified 37 °C, 5% CO_2_ atmosphere incubator.

**Figure A1:**
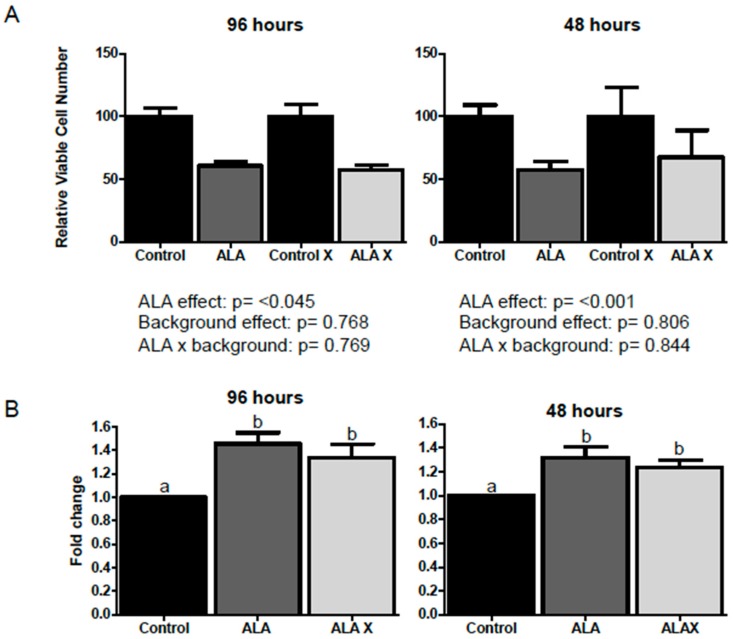
(**A**) Effect of ALA alone or in combination with background fatty acids on cell viability. 100 μM ALA alone or combined with 40 μM each oleic acid (OA) and linoleic acid (LA) both significantly reduced the cell viability of MCF-7 cells following 48 and 96 h treatment. A significant ALA effect (48 h *p* ≤ 0.05; 96 h *p* ≤ 0.001), but no background fatty acid effect was observed. There was no interaction between ALA and background fatty acids at either time point. Cell viability is expressed as a % of the control viable cell number; (**B**) Effect of ALA alone or in combination with background fatty acids on miR-21 expression after 24, 48 and 96 h treatment. This same treatment significantly increased miR-21 expression after the same treatment times. ALA alone and combined with background fatty acids significantly increased the expression of miR-21 at 48 h (fold change = 20–30% increase; *p* ≤ 0.01) and 96 h (fold change = 30–40%; *p* ≤ 0.001) when compared to the control (Figure 1). Since background fatty acids did not influence ALA effect, additional studies were conducted in the presence of background fatty acids to render our findings more physiological. Data were normalized to the endogenous control U6 and fold change was calculated with the 2^−ΔΔ*C*t^ method [19]. Bars with different letters (a,b) are significantly different from one another (*p* < 0.05). X = background fatty acids—oleic acid, linoleic acid.

ALA, EPA, oleic acid (OA), linoleic acid (LA) (all >99% pure) and DHA (>98% pure), were obtained from Sigma-Aldrich. Fatty acid stock solutions were prepared in 100% ethanol and stored at −20 °C [8]. Briefly, fatty acids were reconstituted in charcoal stripped FBS (CS-FBS; Sigma-Aldrich) at a concentration of 4 mM. Appropriate volumes of the fatty acid-CS-FBS solutions required to create desired treatment concentrations and fatty acid ratios were then added to phenol red free DMEM-F12 (Gibco) supplemented with 1% antibiotic-antimycotic, additional CS-FBS to reach a 5% FBS treatment solution, and 1 nM E2 (Sigma-Aldrich) dissolved in ethanol. Fatty acid treatment ratios were based on previous findings described in [5,7]. Mice fed FSO displayed a serum fatty acid ratio of 1.0:0.4:3.1 ALA:EPA:DHA and humans fed FSO displayed a plasma fatty acid ratio of 1.0:1.0:2.5 ALA:EPA:DHA [5,7]. The control treatment medium was equivalent to the treatment medium with the exception of the additional ALA, EPA and DHA; background fatty acids, OA and LA (>99% pure, Sigma Aldrich), were added to the control medium when also contained in the treatment (Appendix Figure A1).

Preliminary experiments were conducted to evaluate the effect of ALA alone or ALA combined with background fatty acids, oleic acid (OA) and linoleic acid (LA), on whether (a) background fatty acids would influence cell viability or miR2-1 expression and (b) the effect observed was due exclusively to ALA, and not simply due to the presence of any fatty acid in the treatment medium (Appendix Figure A1). Background fatty acids did not influence the ALA effect; therefore all studies were conducted in the presence of background fatty acids to render the findings more physiological.

* Note: Experimental treatments from Figure 2 onwards delivered a total dose of 112 μM of n-3 PUFAs (ALA alone or ALA + EPA + DHA), regardless of fatty acid combination, and all treatments also contained background fatty acids. An equimolar concentration of n-3 PUFAs in treatments helped to ensure accurate comparisons.

### 4.2. Trypan Blue Exclusion Assay for Cell Viability

After treatment, cells were detached by trypsinization and, after adding trypan blue, counted using a TC20 automated cell counter (Bio-Rad, Hercules, CA, USA). The total and live cell number for each well was determined and the average viable cell counts of the three wells for each treatment condition (three technical replicates) was divided by the mean of the control wells to present the data as a percentage of the untreated control cell number. By using trypan blue exclusion assay, significant change in viable cell numbers remaining in culture wells at different incubation times with n-3 PUFAs or control conditions was estimated.

### 4.3. RNA Extraction and Real-Time Quantitative PCR (RT-qPCR)

Total RNA was extracted using the mirVana^™^ miRNA isolation kit (Ambion, Life Technologies, Carlsbad, CA, USA), according to the manufacturer’s protocol. RNA concentration and quality were measured using the NanoDrop 2000 Spectrophotometer (Thermo Fischer Scientific, Waltham, MA, USA). For miR-21 analysis, 10 ng of total RNA was reverse transcribed using the TaqMan^®^ microRNA Reverse Transcription kit (Life Technologies, Foster City, MA, USA) and primers specific for miR-21 (Assay ID: 000397) and the endogenous control U6 (Assay ID: 001973) according to the manufacturer’s instructions. RT-qPCR was then performed in triplicate using undiluted cDNA, TaqMan^®^ microRNA assay (20×) and the TaqMan^®^ Universal PCR Master Mix II (2×), no UNG. For mRNA analysis, 2 μg of total RNA were reverse transcribed with random hexamer primers using the High Capacity cDNA Reverse Transcription Kit (Life Technologies) according to the manufacturer’s protocol. qPCR was conducted with the TaqMan^®^ Gene Expression Master Mix (2×) (Life Technologies) and with TaqMan^®^ Gene Expression Assays (*PTEN*: Hs02621230) in triplicate. All qPCR experiments were conducted with 384-well plates and a HT7900 thermocycler (Applied Biosystems, Foster City, CA, USA). Data were normalized to the endogenous control U6 (ID: 001973) (for miR-21) or beta-2 microglobulin (*B2M*) (ID: Hs00984230) (for mRNA) and fold change was calculated with the 2^−ΔΔ*C*t^ method [17].

### 4.4. Protein Biomarker Expression

Total protein was extracted from cells collected as described in [8]. Total protein was separated in a 10% polyacrylamide gel and then transferred to a polyvinylidene difluoride (PVDF) membrane. Primary antibodies included: PTEN (CST 1:1000) and GAPDH (CST 1:4000) (CST, Beverley, MA, USA). Membranes were then incubated with a 1% blocking buffer along with anti-rabbit horseradish peroxidase (HRP)-conjugated secondary antibody (CST, Beverley, MA, USA, 1:4000) for 1 h at room temperature and proteins were detected using LuminataTM Crescendo Western HRP Substrate chemiluminescent reagent (EMD Millipore, WBLUR0100, Billerica, MA, USA). Chemiluminescence was detected on X-ray film (Clonex Corporation, Markham, ON, Canada) and densitometric analysis was performed as described in [7]. GAPDH was used as the loading control after stripping and re-probing with primary antibody for GAPDH. Both technical and biological replicates were carried out in triplicate for cell viability, qPCR and western blot experiments.

### 4.5. Statistical Analysis

Statistical analysis was completed using Graph Pad Prism 5 (Graphpad Software Inc., La Jolla, CA, USA) and Sigma Stat 3.5 (Systat Software, San Jose, CA, USA). All data are presented as mean ± standard error of mean (SEM). For all experiments, significance was set at *p* < 0.05. For cell viability experiments, each treatment group was calculated as % of control and control was either no treatment or + background fatty acids. A two-way ANOVA was conducted to determine the effects of ALA and background fatty acids on cell viability (Appendix Figure A1). Separate one-way ANOVA with post-hoc Tukey test was conducted to analyze the effect of background fatty acid treatments on miR-21 expression for each 48 and 96 h treatments (Appendix Figure A1). Paired *t*-tests were conducted when two groups were being compared, and one-way ANOVA with post-hoc Tukey test was conducted when comparing three or more groups.

## 5. Conclusions

This is the first in vitro study completed on breast cancer cells with the blood ALA:EPA:DHA molar ratios observed in vivo post-ALA consumption. Overall, the results suggest that ALA alone and combined with EPA and DHA at ratios seen in mice and humans post-ALA consumption are able to reduce cell viability and modulate miR-21 expression but they are not directly associated with PTEN gene and protein expressions. The susceptibility of miR-21 to n-3 PUFAs appears to be temporal in nature, and also specific to the type of n-3 PUFA and dosage. Higher DHA concentrations seen in the AnR treatment appear to be most effective at reducing miR-21 expression over the greatest temporal range. Results suggest that time course experiments are crucial when elucidating miRNA-dependent pathways in breast cancer, particularly for clinical applications.

## Abbreviations

ALA	Alpha-linolenic acid
AnR	Animal fatty acid ratio treatment (ALA:EPA:DHA molar ratios in the blood)
BC	Breast cancer
DHA	Docosahexaenoic acid
EPA	Eicosapentaenoic acid
FO	Fish oil
FSO	Flaxseed oil
HuR	Human fatty acid ratio treatment (ALA:EPA:DHA molar ratios in the blood)
miRNA	MicroRNA
n-3 PUFA	Omega-3 polyunsaturated fatty acid

## Appendix A

A significant ALA effect (48 h *p* ≤ 0.05; 96 h *p* ≤ 0.001), but no background fatty acid effect was observed (Figure A1). There was no interaction between ALA and background fatty acids at either time point (Figure A1). ALA alone significantly increased the expression of miR-21 at 48 h (fold change = 20–30% increase; *p* ≤ 0.01) and 96 h (fold change = 30–40%; *p* ≤ 0.001) when compared to the control (Figure A1). Since background fatty acids did not influence the ALA effect, additional studies were conducted in the presence of background fatty acids to render our findings more physiological.
